# Differences in Three Vection Indices (Latency, Duration, and Magnitude) Induced by “Camera-Moving” and “Object-Moving” in a Virtual Computer Graphics World, Despite Similarity in the Retinal Images

**DOI:** 10.1177/2041669520958430

**Published:** 2020-10-15

**Authors:** Hirotaro Sato, Yuki Morimoto, Gerard B. Remijn, Takeharu Seno

**Affiliations:** Faculty of Design, Kyushu University, Fukuoka, Japan

**Keywords:** vection, perception, experimental psychology, camera, tunnel, computer graphics

## Abstract

To create a self-motion (vection) situation in three-dimensional computer graphics (CG), there are mainly two ways: moving a camera toward an object (“camera moving”) or by moving the object and its surrounding environment toward the camera (“object moving”). As both methods vary considerably in the amount of computer calculations involved in generating CG, knowing how each method affects self-motion perception should be important to CG-creators and psychologists. Here, we simulated self-motion in a virtual three-dimensional CG-world, without stereoscopic disparity, which correctly reflected the lighting and glare. Self-motion was induced by “camera moving” or by “object moving,” which in the present experiments was done by moving a tunnel surrounding the camera toward the camera. This produced two retinal images that were virtually identical in Experiment 1 and very similar in Experiments 2 and 3. The stimuli were presented on a large plasma display to 15 naive participants and induced substantial vection. Three experiments comparing vection strength between the two methods found weak but significant differences. The results suggest that when creating CG visual experiences, “camera-moving” induces stronger vection.

## Introduction

When a large visual field is occupied by coherent motion, we can perceive self-motion even though we in fact do not move at all. This illusory self-motion perception induced by visual stimuli is named vection. Vection is ambiguous; often, we cannot discern whether we are moving ourselves or whether only our visual surroundings are moving because the visual input is basically the same. The train illusion is the best example. When you are sitting in a train and the train on the opposite track begins to move rightward, you can perceive that your own train begins to move leftward (Seno & Fukuda, 2012). The train illusion is inevitably ambiguous because both perceptual solutions could be correct. In real life, you can eliminate the ambiguity by looking around the environment where cues exist that can help you determine which perceptual solution is correct (e.g., if the doors of your train are open, it can be a cue that your train is not moving). However, what happens when we need to interpret a situation like the train illusion in a virtual computer graphics (CG) world where no extra cues exist to help you solve the perceptual ambiguity?

In this study, we created two self-motion situations. In one, a camera is moving forward in a large tunnel (the “camera-moving” condition). In the other, the tunnel is moving closer to the camera (the “tunnel-moving” condition). These two self-motion situations are very similar to those causing the train illusion. When we created these situations by using three-dimensional CG (3D CG), the retinal images of both could be virtually identical. However, as will be discussed later, there was a difference between the two in the amount of computer calculations necessary to generate them (i.e., in effective frame rate) and, hence, between processing time and costs. We hypothesized that when the effective frame rate increased and the texture appearance improved due to lower amounts of calculation, as in the camera-moving condition, the obtained vection strength would increase.

Because CG-technology has become more advanced, vection scenes have recently become much more popular and are frequently used in movies and other types of entertainment (e.g., Seno, Murata, et al., 2018; Seno, Tokunaga, et al., 2018; Tokunaga et al., 2016). The future will likely bring even higher quality, large-scale visual presentation techniques, and vection-based virtual experiences will thus likely become even more popular outside the laboratory. For the next generation of CG-based vection technologies, the amount of computer calculations necessary to generate vection scenes is an important issue. To calculate all possible factors when creating a virtual world with CG is impossible because the amount of computer calculations and processing time can become infinite and costly. Thus, CG-creators have searched for ways to reduce these, and the history of CG-research is a history of this process. The development of techniques that reduce computation can be witnessed in creations such as “Environment Mapping” (Greene, 1986), “Metaball” (Ricci et al., 1982), “Marching Cubes” (Lorensen & Cline, 1987), “Real-time cloud rendering” (M. J. Harris & Lastra, 2001), and “Realistic and Fast Cloud Rendering” (Niniane, 2004). This study can also be thought of as an investigation into more efficient and effective CG-expressions of vection.

The CG used in this study were the following. We simulated a virtual 3D space without stereoscopic disparity. In these CG, each texture reflected the lighting in the simulated virtual 3D environment. The visual stimuli covered the large visual field. We also generated stimuli that evoked the perception of moving inside a tunnel by 3D rendering. This motion effect interactively changed with the 3D structures of the simulated world, as did all the lighting effects and geometric appearances. Finally, as colored stimuli were used rather than gray scale stimuli, the simulations very closely mimicked natural vection situations.

The main purpose of this study was to examine whether the virtually identical retinal images that resulted from the “camera moving” and the “tunnel moving” condition could induce vection in totally the same manner or not. In other words, can vection be similarly induced by CG that require different amounts of computer calculations? A small difference in the computer calculations between the two simulations will cause a difference in the effective frame rate and the appearances of the textures. As described in the results, the effective frame rate differed by 4.47% between the camera- and tunnel-moving conditions. We investigated whether this affected vection strength or not.

Related to this purpose, another purpose was to examine how vection could be affected by presenting the observer cues to solve ambiguous situations. Research over the last 45 years has shown that vection can be influenced by many different cues (since Brandt et al., 1973; for a review, see Palmisano et al., 2015; Riecke, 2010), yielding many ways to make effective vection stimuli using CG. For example, vection can be facilitated or inhibited by adding colors to CG (Bonato & Bubka, 2006; Bubka & Bonato, 2010; Seno et al., 2010; Seya et al., 2015). It can also be facilitated by adding jitter or oscillations to CG optic flow (e.g., Bossard et al., 2016, 2020; Bossard & Mestre, 2018; Palmisano et al., 2011; Palmisano & Kim, 2009). The lighting of the CG-world can also modify vection strength (Kim et al., 2016; Nakamura et al., 2010). More basically, increasing the frame rate of CG-vection stimuli increases vection strength (Fujii et al., 2018; Weech et al., 2020). Furthermore, we have examined the material properties of the surface of vection stimuli and found that some material surfaces (e.g., bumpy bark surfaces) are more effective for vection induction than others (e.g., smooth and transparent glass surfaces; Morimoto et al., 2019; Sato et al., 2018a, 2018b). Also, the influence of using new environments like a very large virtual screen (e.g., Mohler et al., 2005) and devices like Oculus Rift (e.g., Palmisano et al., 2017) can modify and highly affect vection strength.

To further investigate the influence of certain cues on vection, in this study, we presented two types of cues of self-motion, consisting of static objects that were put in the virtual space. The objects were a small sphere and an outer box surrounding the tunnel and the camera. In the tunnel-moving condition, the objects did not change, but in the camera-moving condition, the relative size of the objects changed corresponding to their distance to the camera. We here investigated whether the size change of the objects in the camera-moving condition could bias vection.

Three experiments were performed. In Experiment 1, the camera-moving and tunnel-moving conditions were made with two types of tunnels (opaque bark or transparent glass) and movement types (i.e., movement with or without oscillation). The movement was either straightforward or forward with horizontal oscillation. As they were assumed to create identical retinal images, in Experiment 1, we examined whether the camera-moving and the tunnel-moving conditions induced the same vection strengths or not. Because of the difference in the amount of computer calculations to generate the conditions, the frame rate and the appearance of the textures could differ a little. We hypothesized that when the effective frame rate increased and the texture appearance improved, the obtained vection strength should increase. Fujii et al. (2018) and Weech et al. (2020) reported that frame rate and vection strength are positively correlated, and Kim et al. (2016) reported that natural surface stimuli induced stronger vection than unnatural surface stimuli. We confirmed whether these very small differences resulted in a difference in vection.

In Experiment 2, a sphere was placed at the end of the tunnel. The sphere remained in view through the end of the tunnel and could be a standard reference point in space. When the tunnel was moving, the distance between the observer and the sphere was not changed, but when the camera was moving, it gradually became shorter. Only in the camera-moving condition, the expansion of the sphere created additional information: A change in its size could afford the observers with a cue of self-motion. We examined whether this very small difference between the two retinal images could induce a difference in vection or not.

In Experiment 3, the transparent tunnel was put in a larger box with a checkerboard pattern inside. When the camera was moving, the checkerboard pattern gradually changed (i.e., there was motion parallax between glass and the checkerboard and an expansion of the front-wall checkerboard texture), but it was constant when the tunnel was moving. We examined whether this kind of very small change in the background pattern could be a cue of self-motion and induce stronger vection or not.

We hypothesized that in Experiment 1, the camera-moving and tunnel-moving conditions presented to the observers would cause virtually the same retinal images and thus similar vection. However, simultaneously, we hypothesized that when the effective frame rate increased and the texture appearance improved because of the lower amounts of computer calculation in the camera-moving condition, the obtained vection strength would increase.

In Experiment 2, a change in the sphere’s size could present a cue of self-motion in the camera-moving condition. Therefore, even though the resulting retinal images for the two vection conditions could be very similar, the observers could perceive stronger vection in the camera-moving condition than in the tunnel-moving condition. In Experiment 3, the same result could be obtained, in that the changing background could afford the observers with a cue of self-motion causing stronger induced vection in the camera-moving condition than in the tunnel-moving condition. In short, if in the following three experiments no difference can be observed in induced vection between the camera-moving and the tunnel-moving conditions, we could say that any one of these methods is suitable to create CG-vection, and the most cost-effective method can be recommended.

## Methods

### Ethics Statement

The study was preapproved by the Ethics Committee of Kyushu University, and informed consent was obtained from all participants before starting the experiments.

### Apparatus

Stimuli were generated and controlled by a computer (MacBook Pro, Retina, 13 in., Mid 2014) and presented on a plasma display (3D Viera 65 in., Panasonic, Japan, with 1,920 × 1,080-pixel resolution at a 60-Hz refresh rate). The maximum luminance (R, G, B = 255, 255, 255) was 17.3 cd/m^2^. Virtual 3D images were generated using a rendering algorithm from the Unity game development platform (Version 5.6.1f1 Personal). The experiments were conducted in a dark chamber and participants sat in a rocking chair because an unstable sitting condition could enhance vection (Riecke, 2006; Riecke & Feuereissen, 2012).

### Participants

Fifteen adult volunteers (3 females and 12 males; graduate and undergraduate students and paid researchers) participated in all three experiments. All participants were of sound physical and mental health, with normal color vision and eyesight, and no history of any of the following conditions: ear pain or headaches when boarding an aircraft, vestibular system diseases, cardiorespiratory diseases, moderate balance disorders, dizziness, or altitude sickness. No participants were aware of the purpose of the experiments. Average age was 26.8 ± 9.2 years (range: 21–53 years).

### Stimuli

The stimuli subtended 100.2° × 71° in visual angle at a 57-cm viewing distance. The duration of stimulus presentation was fixed at 30 s, and the frame rate was 60 frames per second. We simulated a 20 m × 20 m × 230 m (width, height, and length) tunnel. The simulated speed of the straightforward self-motion was 4.8 m/s. The field of view was 44.4°. For the background, we used Skybox Blue Nebula from the Unity Asset Store. This background shows the void of space with an infinite distance. This means that the background did not change either in the camera-moving or the tunnel-moving condition.

**Figure 1.**
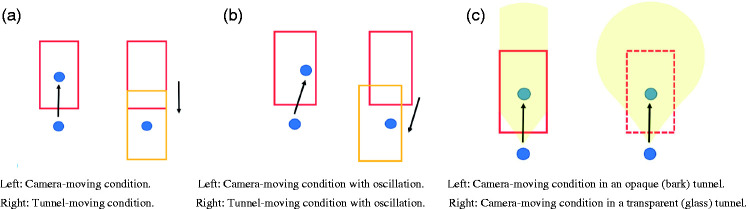
Schematic Illustration of the Stimuli in Experiment 1, the View From Above. A: The difference between the camera-moving (left) and tunnel-moving (right) conditions. The red rectangle indicates the tunnel and the blue circle is the camera, that is, the self. When the tunnel moves forward to the self (the translation of the rectangle) or when the self moves forward to the tunnel (the translation of the blue circles), forward self-motion can be created and the retinal images of these two are virtually identical. B: With horizontal oscillation of the camera (left) and tunnel-moving (right). The oblique black arrows indicate that either the camera or the tunnel moves in an oblique direction. These two are both moving with horizontal oscillations. C: The areas painted in yellow indicate the visible areas for bark (left) and glass (right) tunnels. In the bark condition, the participants could see just the inside of the tunnel, but in the glass condition, they could see the outside of the tunnel as well.

**Figure 2.**
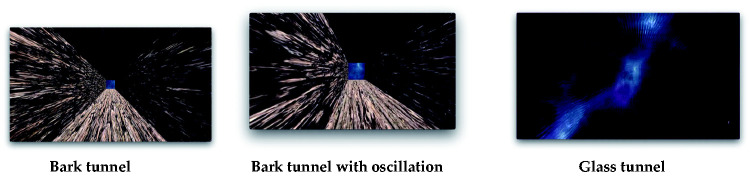
The stimuli used in experiment 1. top: the inside of the bark tunnel (opaque). Middle: the inside of the bark tunnel (opaque) when the camera or the tunnel moved in oblique direction because of the oscillation. Bottom: the transparent glass tunnel. The background can be seen through the glass, which had a bumped texture (please also see the movies in http://senotake.jp/demo.html).

The two conditions (camera-moving or tunnel-moving) were created by inputting the script for the motion (speed) to either the camera or the tunnel (Figure 1). For the opaque tunnel, we used a virtual surface material made from “bark” (Figure 2, left), because this virtual material induced the strongest vection in our previous study (Morimoto et al., 2019; Sato et al., 2018a, 2018b). For the transparent tunnel (Figure 2, right), we used a “glass” surface material. To create them, we used Assets (ADG Textures Bark c2, MKGlassFree Glass2) from Unity. For this manipulation, the properties of the surface materials were not important. Rather, the importance was in the fact that there were opaque (bark) and transparent (glass) tunnel conditions. In the transparent condition, the participants saw through the background, which they could not in the opaque condition.^1^ The lighting was set as ambient. Every portion of the created space was lighted by the same, equal lighting, so there was no single light-emitting spot. Thus, even the inside of the tunnel could be seen by the participants. In both conditions, the same bumped texture was added to the surface. Because of this bumped surface, even when the transparency of the glass was set as 100%, the glass walls could be seen by the simulated reflected light. The width of the horizontal oscillation was 5 m, and the speed was 2 m/s. The oscillations followed sinusoidal reciprocating motion. The speed of the forward motion component was always set as constant, that is, 4.8 m/s. Thus, the motion speed in the oscillation conditions was relatively faster than in the no-oscillation conditions, that is, 5.2 m/s. It has been repeatedly reported that oscillatory motion can enhance vection, and it can also improve the observers’ perception of the distance traveled in comparison with a purely translational flow (e.g., Bossard et al., 2016, 2020; Bossard & Mestre, 2018; Palmisano et al., 2011; Palmisano & Kim, 2009). The assumed underlying reason of this facilitation effect of oscillation on vection is that a changing pattern of retinal motion can serve to reduce visual adaptation and thus maintains a sustained sensitivity to optic flow (Bossard & Mestre, 2018; Kim & Khuu, 2014). Oscillation increased the amount of computer calculation and thus, when we think about the possible application of our study, this kind of additional computer calculation should be considered. In total, there were eight conditions: 2 Motion Types (camera-moving or tunnel-moving) × 2 Tunnels (opaque “bark” or transparent “glass”) × 2 Oscillation Types (with or without oscillation).

**Figure 3.**
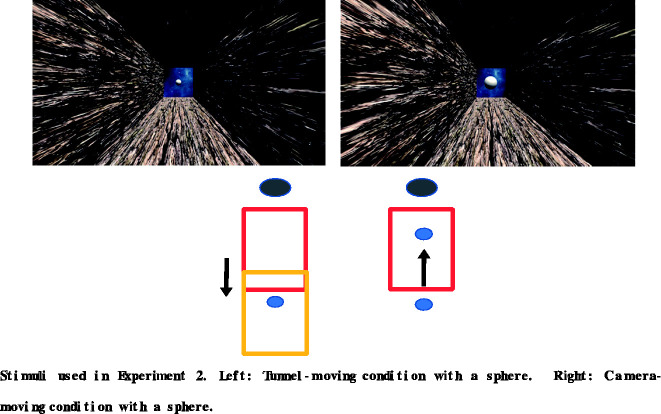
Top: the stimuli used in experiment 2. Left: in the beginning of the movie of either motion condition, the sphere was relatively small. right: compared with the tunnel-moving condition, however, at the end of the movie of the camera-moving condition, the size of the sphere was somewhat larger than in the beginning. Bottom: schematic illustrations of the motion conditions in experiment 2. The blue circle is the camera, the rectangle is the tunnel and the black bigger circle is the sphere placed at the exit of the tunnel.

In Experiment 2, a simple white sphere without any specific meaning was placed at the end of the tunnel (Figure 3). The diameter of the sphere was 10 m. The retinal image of the sphere became larger when the camera moved toward it but was constant in the tunnel-moving condition (Figure 3). In the camera-moving condition, the observers thus came closer to the sphere, whereas in the tunnel-moving condition, only the tunnel’s position changed and the observers and the sphere were static and did not change their relative positions. Therefore, in the camera-moving condition, the size of the sphere should change, and in the tunnel-moving condition, it should not. For enhancing the difference between camera- and tunnel-moving conditions, we put this sphere. This small difference might cue the participant as to which was moving, the self or the tunnel. In this experiment, we only used the bark surface material for the tunnel. As in Experiment 1, we also examined the effect of the horizontal oscillation. Thus, there were four stimuli (2 Motion Types, i.e., camera-moving or tunnel-moving × 2 Oscillation Types).

**Figure 4.**
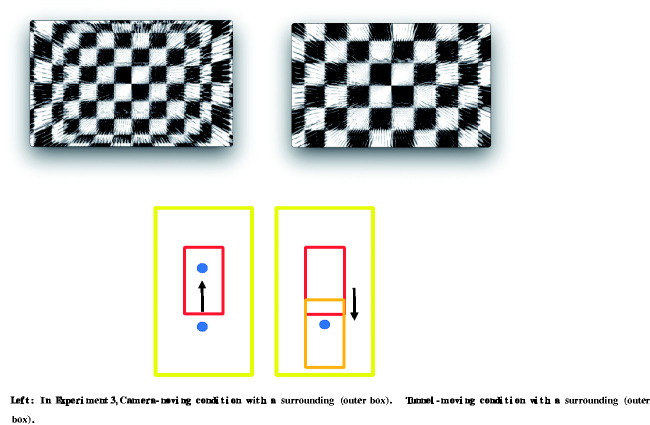
Top: An example of the stimuli used in experiment 3. The starting point (left) and the finishing point (right). The bumped glass could be seen even though it was 100% transparent, because of the reflection of the ambient light. The surrounding environment, that is, the larger surrounding box with checkerboard squares inside, could be seen too. The size of the checkerboard squares differed in the camera-moving condition but not in the tunnel-moving condition. Bottom: schematic illustrations of the motion conditions in experiment 3. The yellow outer box surrounding the camera and the tunnel is the background painted in checkerboard squares.

In Experiment 3, the tunnel made of glass was put into a larger box with a checkerboard pattern inside (Figure 4). The box was 400 m × 300 m × 400 m (width, height, and length). The shader for Unlit Checkerboard in Unity was used to make this condition. Participants could perceive the motion of the background when the camera was moving, but not when the tunnel was moving, because the size of the squares were enlarged as progress was made through the tunnel. There was just one big difference, that is, camera- versus tunnel-moving. In the tunnel-moving condition, the effective frame rate was lower than in the camera-moving condition. Also, there were differences in the motion parallax cue and the expansion cue between the two conditions. These differences could afford a hint of self-motion or object motion for the observers. We tested whether these small differences affected the obtained vection strength or not. Only the glass tunnel was used in this condition. We again examined the effect of horizontal oscillation. Thus, there were four stimuli (2 Motion Types, i.e., camera-moving or tunnel moving, × 2 Oscillation Types).

**Figure 5.**
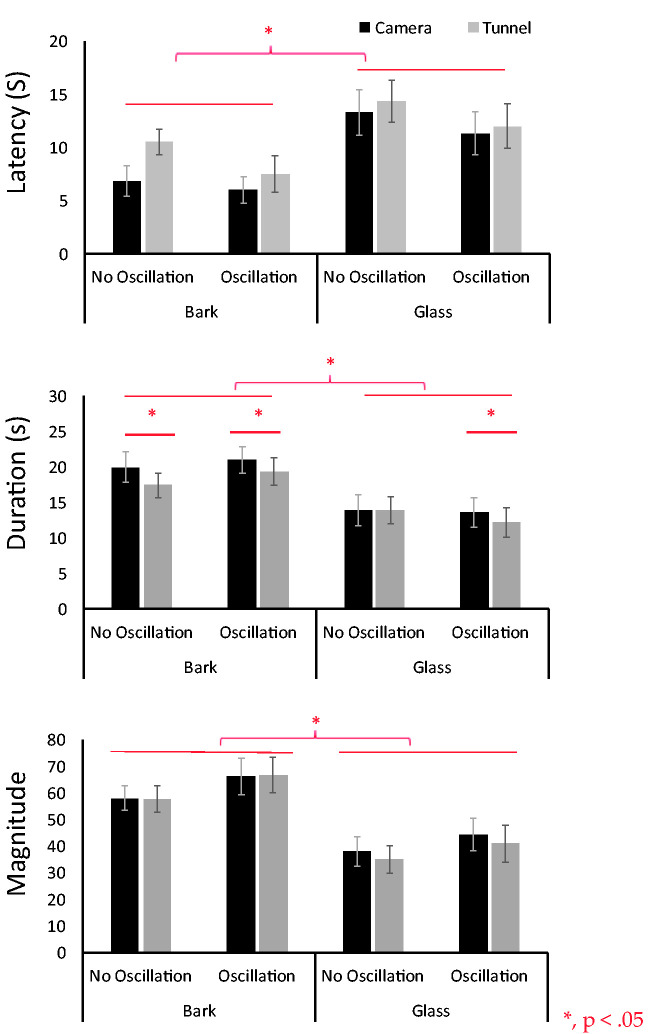
The results of experiment 1. Three vection indices are indicated, that is, latency (top), duration (middle), and magnitude of vection (bottom). The horizontal axis indicates eight stimulus conditions. Black bars and gray bars are camera- and tunnel-moving conditions, respectively.

**Figure 6.**
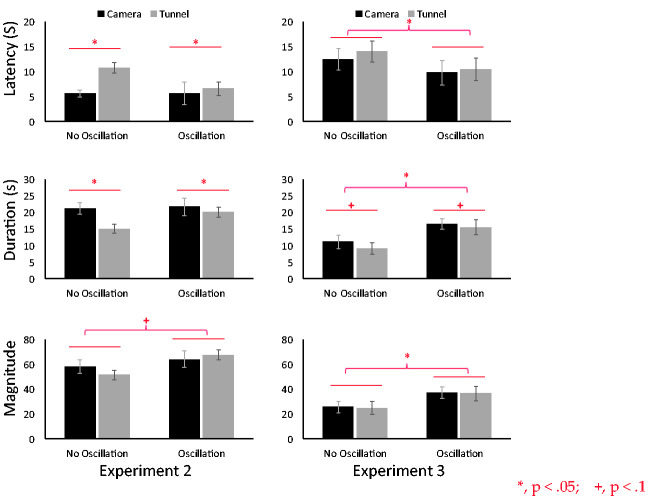
The results of experiments 2 and 3. Three vection indices are indicated, that is, latency (top), duration (middle), and magnitude of vection (bottom). The horizontal axis indicates four stimulus conditions. black bars and grey bars are camera- and tunnel moving conditions, respectively. The panels on the left and on the right are the results of experiment 2 and 3, respectively.

### Procedure

There was no fixation point and participants’ heads were not fixed by a chinrest. In each trial, the stimulus was presented for 30 s. Participants were asked to press the space key whenever they perceived forward self-motion and to keep the key depressed for the duration of the perception. We recorded the latency and duration of vection. After each trial, participants rated the subjective vection strength using a 100-point rating scale in which 0 represented *no vection* and 100 represented *very strong vection*. These procedures have been used in our previous studies (Seno et al., 2014, 2017). Each stimulus condition was presented 2 times in Experiment 1 and 3 times in Experiments 2 and 3. Experiment 1 had 16 trials (8 Conditions × 2 Repetitions), and Experiments 2 and 3 had 12 trials each (4 Conditions × 3 Repetitions). The condition order was randomized.

## Results and Discussion

### Experiment 1

The results were shown in Figure 5. A three-way analysis of variance (ANOVA) revealed a main effect for tunnel type, (bark or glass) in all three vection indices, latency: *F*(1, 14) = 16.91, *p* = .001; duration: *F*(1, 14) = 25.72, *p* = .0002; magnitude: *F*(1, 14) = 33.51, *p* = .0000. Latency was shorter for bark, duration was longer and magnitude larger, thus showing stronger vection overall in the bark condition. There was also a main effect of motion type (camera-moving or tunnel-moving) for duration, *F*(1, 14) = 9.17, *p* = .009, but not for latency, *F*(1, 14) = 2.19, *p* = .16, and magnitude, *F*(1, 14) = 1.50, *p* = .23. Duration was longer in the camera-moving condition. We did not observe a significant main effect of oscillation type (with or without oscillation), latency: *F*(1, 14) = 2.39, *p* = .14; duration: *F*(1, 14) = .39, *p* = .53; magnitude: *F*(1, 14) = 1.67, *p* = .21. Vection latency, duration, and magnitude were the same between with and without oscillation. An effect of oscillation on vection thus was not observed. We also did not observe any significant interactions.

### Experiment 2

The results were shown in Figure 6. A two-way ANOVA revealed a significant main effect of motion type (camera-moving or tunnel-moving) for vection latency and duration, but not for magnitude, latency: *F*(1, 14) = 13.13, *p* = .002; duration: *F*(1, 14) = 12.96, *p* = .002; magnitude: *F*(1, 14) = 0.15, *p* = .69. Latency was shorter, and duration was longer in the camera-moving condition, indicating that stronger vection was obtained in the camera-moving condition. We found a tendency for oscillation type to affect magnitude but not latency or duration, latency: *F*(1, 14) = 2.63, *p* = .12; duration: *F*(1, 14) = 2.42, *p* = .10; magnitude: *F*(1, 14) = 4.15, *p* = .06. Magnitude was larger in the oscillation condition. We thus at least partially succeeded in replicating the oscillation advantage in facilitating vection in this experiment. Again, we did not observe any significant interactions.

### Experiment 3

The results were shown in Figure 6. A two-way ANOVA revealed a tendency for motion type (camera-moving or tunnel-moving) to affect duration but not the other two measures of vection, latency: *F*(1, 14) = 0.52, *p* = .47; duration: *F*(1, 14) = 3.80, *p* = .07; magnitude: *F*(1, 14) = 0.15, *p* = .69. Duration was longer in the camera-moving condition. Based on this, we could say that to some extent stronger vection was obtained in the camera-moving condition. We found a main effect of oscillation type on all three vection indices and thus a strong advantage of the oscillation in facilitating vection was replicated, latency: *F*(1, 14) = 12.58, *p* = .003; duration: *F*(1, 14) = 10.43, *p* = .006; magnitude: *F*(1, 14) = 6.09, *p* = .02. Again, we did not observe any significant interactions.

In Experiment 1, vection was stronger for the bark tunnel than for the glass tunnel. This agrees with our previous findings (Morimoto et al., 2019; Sato et al., 2018a, 2018b). Motion type (camera- or tunnel-moving) only significantly affected the duration of vection: camera-moving induced longer lasting vection than tunnel-moving. In this experiment, however, the two retinal images of the two motion types should have been totally the same (identical) because ambient light was used and the background was infinite. Thus, in this respect, there should have been no difference between the two. It is possible, however, that this significant main effect of motion type (camera- or tunnel-moving) on duration is related to the fact that these two methods require different amounts of calculations by the computer when generating the CG.

By assuming that there is a direct relation between CG-related calculations and effective frame rate, we therefore compared these by means of an application (Unity profiler; http://tsubakit1.hateblo.jp/entry/2016/05/09/073000). The results showed that the effective frame rate for the camera-moving condition was 50.01 frames per second and for the tunnel-moving condition 47.87 frames per second. For generating our stimuli, camera moving thus was 4.47% smoother than tunnel moving, and Fujii et al. (2018) and Weech et al. (2020) indeed reported that the higher the frame rate, the stronger the vection becomes. This could be a reason why for the duration index in Experiment 1 vection became stronger in the camera-moving condition, even though the retinal images of the two conditions must have been almost identical. However, we should note here clearly that the physical difference between the two might not be sufficiently large enough to create a difference in vection strength. This should be considered carefully as a possible caveat for further discussion.

Other unknown factors in the system (Unity) may have contributed to the difference, however, what for now can be said is that as regards computer programming, the camera-moving method was more natural and simple than the tunnel-moving method. Because of this simplicity and naturalness, camera moving may have rendered more realistic vection. It has been repeatedly reported that more natural stimuli can induce stronger vection than less natural ones (e.g., Kim et al., 2016; Nakamura, 2013; Riecke et al., 2006; Schulte-Pelkum et al., 2004). However, there was no difference in the other vection indices (i.e., latency and magnitude), so the “naturalness” claim might be weak here and more research is needed.

In Experiment 2, we confirmed a weak advantage of using the oscillation but only on vection magnitude. As for the two motion conditions, significantly stronger vection was induced by the camera-moving condition than by the tunnel-moving condition for vection latency and duration. Even though the retinal images between the two conditions were very similar, the moving camera was a more effective inducer of vection. Adding the sphere did not make a big difference between the retinal images in the two motion types. Only in the camera-moving condition an expansion of the texture of the sphere occurred. In fact, in the after-experiment debriefing session, only one out of 15 participants reported orally and explicitly that he actually noticed the size change in the sphere. However, it is nevertheless possible that the change significantly affected vection latency, which would suggest that at the very early stage of stimulus presentation (within a few seconds), the small change could have facilitated vection. This cue could have made vection in the camera-moving condition begin earlier and may have been efficiently used to discriminate the moving camera (self) from the moving tunnel (object).

Here, we should note that if the sphere was assumed to move with the camera (self) at the same speed in depth, the distance between the sphere and the observer should be constant even if the camera was moved. If the participants assumed this situation, the sphere should not have facilitated vection. The most plausible explanation is that the participants may have used their knowledge about the world, knowing that in the natural world self-movement (camera) toward a static object is more natural than an object moving in the same manner as the camera (self). Although this is just speculation, we might be able to say that our vection experience will be biased by the inference that a sphere (an object) should be static and that a change in its size should indicate self-motion (camera-moving) rather than object motion (tunnel-moving).

Despite being reported in past studies (Bossard et al., 2016; Bossard & Mestre, 2018; Palmisano et al., 2011), we could not confirm any advantage of using horizontal oscillation in Experiment 1. However, we were able to see such effects in Experiments 2 and 3. Especially in Experiment 3, we confirmed a very strong advantage of using horizontal oscillation on all three vection indices. The reason why we could not get the same effect of oscillation in Experiment 1 might be the following. Apthorp and Palmisano (2014) reported that the oscillation advantage on vection was eliminated when the perceived speed of motion in depth was matched between the simple pure radial flow and the same flow with oscillation. Bossard and Mestre (2018) also pointed out that the effect of oscillation on the correctness of the perceived and estimated distance in the virtual space is related to the perceived speed of self-motion. Redlick et al. (2001) and Harris et al. (2012), respectively, also reported that the distance estimation during vection is highly affected by the simulated speed of self-motion. Thus, the difference of the perceived and simulated speeds between nonoscillated and oscillated motion conditions might be the important factor. For inducing the facilitation effect of the oscillation, the perceived speed should be faster in the oscillated condition than in the pure radial flow.

In this study, the same tunnel was used with a fixed length in depth of 230 m in the virtual space. The participants could have assumed that the tunnel’s length was always the same between the motion conditions because the exit of the tunnel could have afforded a cue to its length. That is, the speed of motion in depth was always the same, and the participants could have inferred that the same length in depth was moved in the same amount of time (30 s). This meant that the perceived speed in depth should have always been the same. Following this, if the perceived speed was the same, then the oscillation’s facilitation effect on vection should have been very weak, as Apthorp and Palmisano (2014) suggested. It should be noted, though, that Bossard et al. (2020) reported that viewpoint oscillations did not improve the accuracy of estimated traveling distance of participants who were walking on a treadmill under a visual condition simulating self-motion in depth. Following this, the advantage of the oscillation on self-motion perception could be affected easily by some other factors like multi-modal inputs or cognitive loads. Thus, there may be other possible reasons for the absence of a strong effect of oscillation on vection in Experiment 1. We should further examine this in the next study.

Once again, also in Experiments 2 and 3, stronger vection was induced by the camera-moving condition than by the tunnel-moving condition but mainly for vection duration. In Experiment 1, we also saw an effect only on duration. This suggests that vection duration is more sensitive to how vection is created (moving the camera vs. moving the tunnel) than are the other indices.

In Experiments 2 and 3, retinal images were very similar and the camera-moving and tunnel-moving conditions should have induced very similar vection. However, although the effect was not very strong, we were able to find a certain difference between the two motion conditions that could be emphasized when visual cues allowed participants to know that the camera was moving. Therefore, we can say that there is some merit to using camera-moving (self-moving) CG for effective vection induction rather than using object-moving CG.

Related to camera moving, Nakamura (2013) proposed the “naturalness hypothesis, pp. 66–69” in vection, saying that vection is stronger for more natural images. A lot of data in many previous vection studies have provided evidence for this hypothesis (e.g., Allison et al., 1999; Bubka & Bonato, 2010; Kim et al., 2016; Klient, 1937; Lishman & Lee, 1973; Riecke et al., 2006; Schulte-Pelkum et al., 2004; Telford et al., 1995; Witkin & Asch 1948;). For example, Bubka and Bonato (2010) compared videos shot either from a smooth rolling cart or with a handheld camera that yielded gait information in addition to global expansion. Their results showed that vection latency was faster, and magnitude was larger during actual self-motion with a handheld camera. They clearly showed that the visual-field features that are common during actual self-motion can enhance vection even in a virtual environment. The same results were reported by Lécuyer et al. (2006). They reported that when the camera motion mimics how our body and eyes move naturally when walking in real life, the camera motion could improve vection. The results in this study obeyed this rule again, we think. Camera-moving is more natural for vection induction than tunnel-moving, even when a virtual space and a virtual CG situation are created.

Our results may also shed light on cybersickness in virtual reality (VR) situations. Hu et al. (2019) reported that the characteristics of the virtual camera movement (e.g., translational acceleration and rotational velocity) and the composition of the virtual environment (e.g., scene depth) contribute to perceived discomfort, that is, motion sickness. Palmisano et al. (2017) also reported that cybersickness could be enhanced by adding oscillation to the radial flow. In this study, without our intention, we may have incidentally shown that prior knowledge about the length of the virtual self-motion in depth, even with the oscillations, might reduce vection and related cybersickness. When driving a car through a long tunnel, a lot of people have problems with steadying the visual image because there is no clear focus point at the end. However, if they know the length of the tunnel (or how long they need to be driving still), this kind of sickness might be reduced. More studies are needed in the future.

As described in the “Introduction” section, searching for more economical ways to reduce calculations is a very important topic in CG-research (e.g., “Environment Mapping,” Greene, 1986; “Real-time cloud rendering,” Harris & Lastra, 2001; “Marching Cubes,” Lorensen & Cline, 1987; “Realistic and Fast Cloud Rendering,” Niniane, 2004; “Metaball”, Ricci et al., 1982). In this study, we examined the difference between two ways in which CG can be used to create vection. The difference was not large, but there were certain effects that could be emphasized by additional visual cues. We hope that designers will use this knowledge for making more attractive and stronger vection movies in the future.

## Conclusion

When creating vection stimuli in CG-worlds, moving the camera (viewpoint) and moving the surrounding environments by using object-motion (tunnel) can have a different effect on vection induction. The present results seem to suggest that camera-moving generally generates more profound vection. However, it should be noted here that there is a limitation to this conclusion because there might be other variables involved in the stimuli. This should be further examined in the future.

## Note

1 We applied the built-in shader from Unity to the materials. The shader incorporated physically based shading, simulating the interactions between materials and light in a way that mimics reality. The shader followed principles of physics, including energy conservation, Fresnel reflections, and how surfaces occlude themselves. This shader is suitable for hard surfaces such as bark and glass. Further details of the surface quality rendering can be found in the documentation on the Unity website (https://docs.unity3d.com/Manual/shader-StandardShader.html).
